# ccPDB 2.0: an updated version of datasets created and compiled from Protein Data Bank

**DOI:** 10.1093/database/bay142

**Published:** 2019-01-23

**Authors:** Piyush Agrawal, Sumeet Patiyal, Rajesh Kumar, Vinod Kumar, Harinder Singh, Pawan Kumar Raghav, Gajendra P S Raghava

**Affiliations:** 1 Bioinformatics Center, CSIR-Institute of Microbial Technology, India; 2 Department of Computational Biology, Indraprastha Institute of Information Technology, Okhla Industrial Estate, Phase III, New Delhi, India; 3 J. Craig Venter Institute 9605 Medical Center Drive, Suite 150 Rockville, MD, USA

## Abstract

ccPDB 2.0 (http://webs.iiitd.edu.in/raghava/ccpdb) is an updated version of the manually curated database ccPDB that maintains datasets required for developing methods to predict the structure and function of proteins. The number of datasets compiled from literature increased from 45 to 141 in ccPDB 2.0. Similarly, the number of protein structures used for creating datasets also increased from ~74 000 to ~137 000 (PDB March 2018 release). ccPDB 2.0 provides the same web services and flexible tools which were present in the previous version of the database. In the updated version, links of the number of methods developed in the past few years have also been incorporated. This updated resource is built on responsive templates which is compatible with smartphones (mobile, iPhone, iPad, tablets etc.) and large screen gadgets. In summary, ccPDB 2.0 is a user-friendly web-based platform that provides comprehensive as well as updated information about datasets.

## Introduction

Advancement in the sequencing technology has enormously increased the protein sequence information in the databases. This increase in protein sequence data has widened the gap between the sequences and annotations (1). Therefore, to fill this gap *in silico* tools are required for annotating the function of these proteins since it is very cumbersome to obtain a crystal structure of all the protein sequences. Therefore, to combat this situation, the number of sequence-based tools has been developed in the past few decades (1–16). These methods are developed using the experimentally proven structural information present in the Protein Data Bank (PDB) (17). Hence, cleaned and refined datasets are required for training, testing and validation of the new method and for benchmarking previous ones.

In order to facilitate scientific community working in the field of structural biology, we created a database ccPDB (18) in 2011, where we have collected and compiled the experimentally validated datasets from the literature. In addition, we provide a web-based platform that allows users to create customized datasets as per their requirement from the PDB July 2011. In the past 7 years, the number of structures in PDB has been nearly doubled, as PDB is continually growing over the years. In addition, many methods which use datasets derived from protein structures also increased drastically. There is a significant increase in the number of datasets and the number of protein structures used to create these datasets. In order to provide updated information to the structural biologist, we have developed ccPDB 2.0 which is an updated version of ccPDB.

## Materials and methods

### Data collection and organization

Since the first version of ccPDB (18) published in 2011, there has been enormous growth in the development of improved methods in the field of secondary structure prediction (9, 19–24), irregular secondary structure prediction (10, 25–28), protein–ligand interactions (7, 15, 16, 29), DNA/RNA–protein interactions (13, 30, 31), protein crystallization and propensity prediction (32–35), dihedral angle prediction (6, 36–38), surface accessibility prediction (39), Rotamer libraries (8) and others (40–43). These methods have been found to annotate protein structure and function in comparison to earlier methods. Therefore, we have performed a major update on the data developed for annotating protein structure and function in the past 7 years. We collected the experimentally validated datasets published in the literature for developing different prediction methods. These datasets were extracted from the PubMed articles and their supplementary materials, websites, databases or directly from the authors.

In order to create datasets, we downloaded all the PDB files till March 2018 release from RCSB-PDB (http://www.pdb.org/) (44) and maintained/mirrored these PDB files at our server. We also maintained DSSP (45, 46) and other PDB-related information at our server. Therefore, a user can create its own customized datasets using these files. Also, we used different software for generating useful information from PDB files. Some of the software includes the following: (i) PROMOTIF (47) for identifying different structural motifs, (ii) LPC (48) for generating protein–ligand interaction data, (iii) HBPLUS (49) for generating protein–DNA/RNA interaction data, (iv) BlastClust for generating protein cluster based on sequence similarity and (v) in-house PERL and Python scripts for analyzing PDB files and different calculations.

Broadly, datasets created/compiled in ccPDB can be divided into two categories: (i) datasets for structure/function annotation at the protein level and (ii) datasets for annotation of protein at the residue level. In the case of structural annotation at the protein level, the overall function of a protein is estimated like the prediction of ATP-, RNA- and DNA-binding proteins. In case of residue level annotation, we predict the function of each residue in a protein, like the prediction of ATP-, RNA- and DNA-interacting residues in a protein.

## Database architecture

A ccPDB 2.0 back end is built using Apache HTTP server 2.2 and MySQL server 5.1.47 and front end using HTML, PHP 5.2.9 and JAVA scripts. We used an HTML5 web template for making a website compatible with mobile and tablet. The abovementioned technologies were used as they are platform independent and open source.

## ccPDB 2.0 implementation

ccPDB 2.0 is an updated and comprehensive database which maintains existing datasets obtained from the published literature and datasets derived from the PDB files. Besides, ccPDB 2.0 also allows a user to create its own customized datasets using PDB’s latest data. Functioning of the database can be broadly classified into three major sections. Details of these sections are mentioned below.

### Collection and compilation of datasets

**Figure 1:**
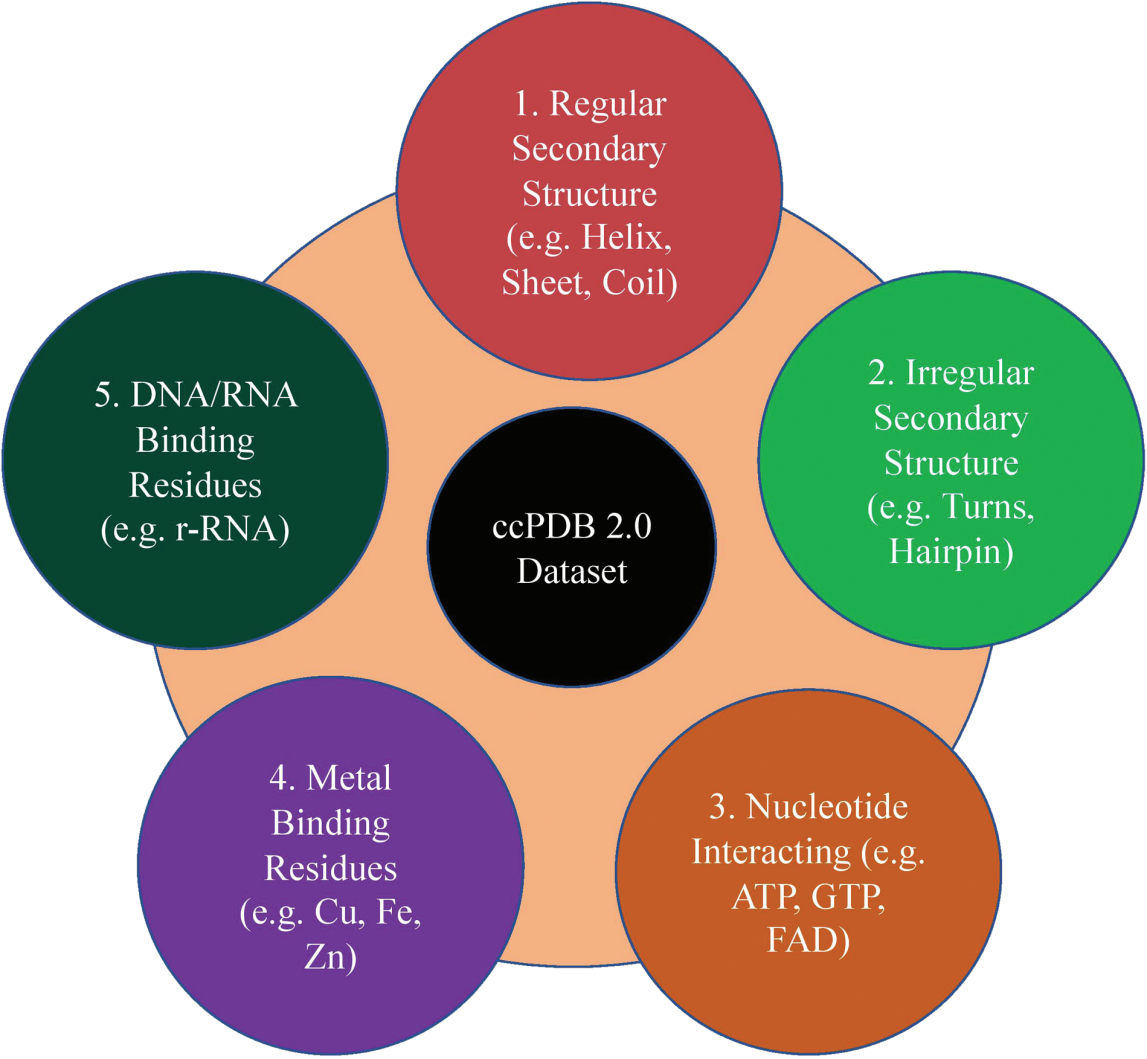
Dataset types present in ccPDB 2.0.

This section maintains the experimentally validated and published datasets collected from the literature after an extensive search. This section was present in the previous version too. In the current database, we have included the datasets published in the past 7 years (Figure 1). We have added some datasets which were not included in the previous version like metal-interacting residue datasets, antigen–antibody interaction datasets.

### Creation of datasets

**Figure 2:**
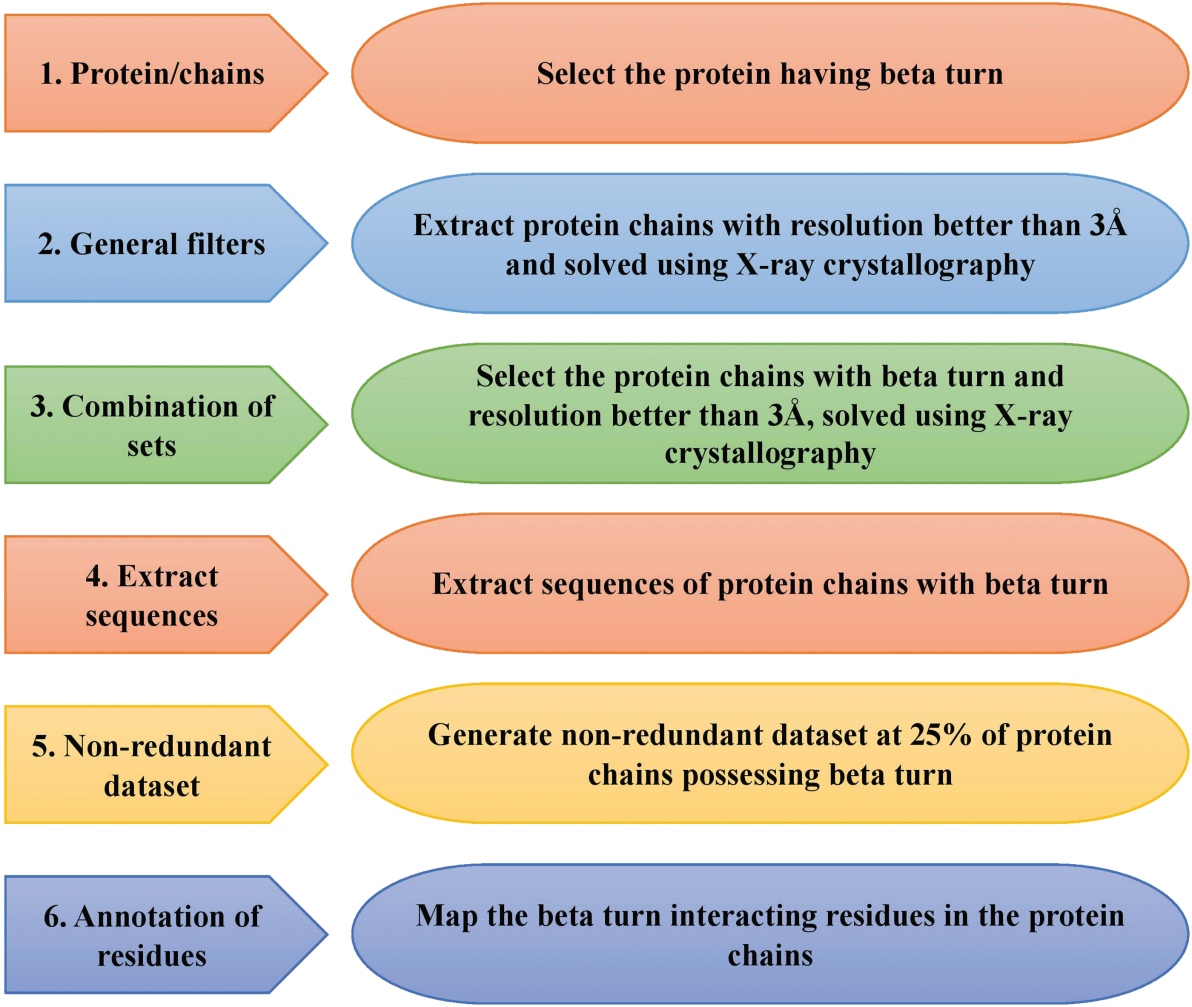
Schematic representation of steps of data set creation module of ccPDB 2.0.

This is an important module of the ccPDB database that allows users to create customized dataset as per their requirement. This module enables the user to create any type of dataset from the latest release of PDB (March 2018 release). This kind of dataset is very useful for benchmarking different methods and developing new method as the performance of a method largely depends on the dataset size. Different type of datasets compiled from PDB is listed in Figure 1 along with their brief compilation procedure. In order to create a new or customized dataset, the user needs to perform the six steps as explained in Figure 2.

**Figure 3:**
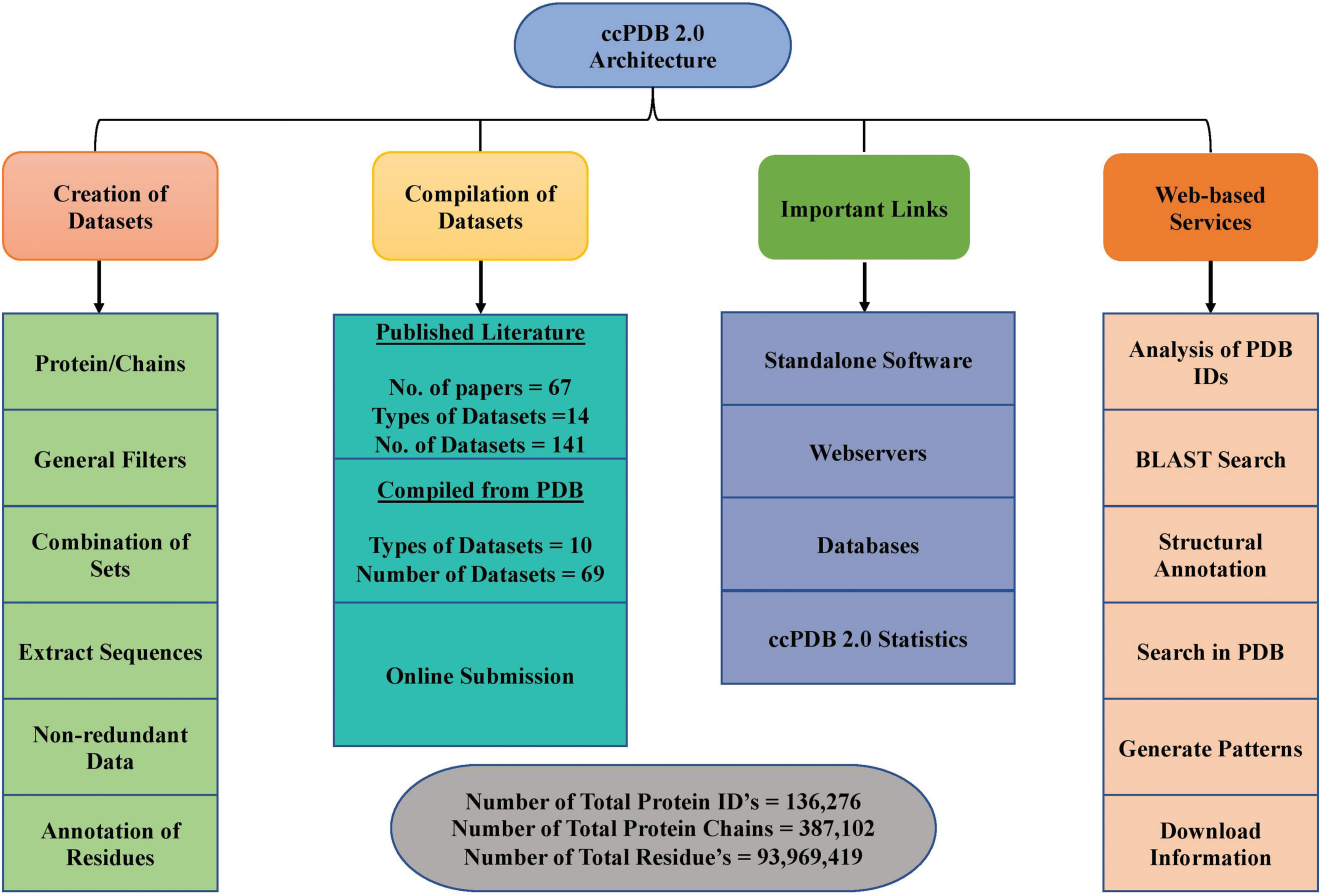
Architecture of ccPDB 2.0.

### Web services and availability

ccPDB 2.0 is freely available at http://webs.iiitd.edu.in/raghava/ccpdb/. ccPDB 2.0 provides the same web services which were earlier present in the first version of this database. The website is compatible with different platforms like desktop, smartphone and tablet. We have also retained the previous version of the ccPDB which is maintained at http://crdd.osdd.net/raghava/ccpdb/.

## Results and discussion

The ccPDB 2.0 database is an updated version of ccPDB which was created to facilitate the user to access the latest information related to protein structural annotation and function. The first version of ccPDB contains very few datasets which were collected from the literature. In the previous version there was a total of 45 datasets compiled from 37 studies. However, the updated database maintains information of 141 experimentally validated datasets compiled from around 68 studies. There were a total 407 200 chains present at the protein level and 1 928 972 chains at residue level in comparison to 340 864 and 6904 chains at protein and residue level, respectively, present in the previous version. We added a new data type `Metal and Ions Interacting Residue’ which was not there in the earlier version.

In the first version of ccPDB, there were only two datasets for regular and nine for irregular secondary structure prediction whereas in the updated version there are 10 and 13 datasets for regular and irregular secondary structure prediction, respectively. The number of datasets for protein–nucleotide interaction in the first version was four; however, in the updated version it has changed to 22. We also included 13 protein–metal ions and protein–acid radical ligand dataset in the updated version which was previously not present. In the previous version, the dataset number of DNA/RNA-interacting proteins at protein level was 5 and 7 at residue level whereas in the updated version it has increased to 14 and 13, respectively. Likewise, in the updated version dihedral angle prediction dataset has risen from 1 to 20, protein crystallization dataset has increased from 3 to 12, bacterial protein interaction dataset has increased from 4 to 6 and surface accessibility prediction dataset has increased from 1 to 3. This tremendous growth of datasets in the literature clearly shows the importance of protein structural annotation and function and how it could lead to better understanding of the role of proteins in various biological and cellular processes.

In order to provide ready-to-use datasets for developing new prediction methods, we compiled information of 70 customized datasets from PDB using standard protocols. Datasets were assembled using the latest PDB March 2018 release which consists of ~137 000 PDB tertiary structures which is nearly double to the files present in the first version of ccPDB which consists of around 75 000 PDB structures. Due to an increase in the number of PDB structures, there was a considerable growth in the number of chains in the updated version. In the previous version, there was a total 17 731 chains at the protein level and 66 368 chains at residue level whereas in the updated version the number of chains at protein level increases to 33 488 and at residue level it changed to 139 902. The number of protein chains for regular secondary structure has been increased from 5877 to 17 608. Likewise, for irregular secondary structure there was an enormous increase in the number of protein chains for different beta turns, gamma turns, psiloop and hairpin. DNA- and RNA-interacting protein chains were increased to 560 and 415 from 417 and 282, respectively, in the updated version.

We also observed an increase in the number of ligand-interacting proteins and metal-interacting proteins in the updated version of ccPDB. To assist the scientific community, we created the dataset for some of the widely used protein-interacting ligands and metals. A user can download these datasets by clicking on the desired dataset present at our website http://webs.iiitd.edu.in/raghava/ccpdb/collect.php. Comparison of the statistics between ccPDB and ccPDB 2.0 is given in Table 1.

**Table 1:** Comparison of datasets compiled from literature and created using PDB at ccPDB and ccPDB 2.0

**Sr. No.**	**Type of dataset**	**Description**	**ccPDB** **(No. of protein chains)**	**ccPDB 2.0** ^*^ **(No. of protein chains)**	**Dataset compiled from the literature in ccPDB (no. of protein chains)**	**Dataset compiled from the literature in ccPDB 2.0** **(no. of protein chains)**
1	Secondary structure	Eight state	5877	17 608	2 (919)	10 (30 426)
		Three state	5877	17 608		
2	Irregular secondary structure	Beta turn I	6691	16 195	9 (5045)	13 (1 913 701)
		Beta turn I’	5424	7070		
		Beta turn II	2324	12 429		
		Beta turn II’	6618	5393		
		Beta turn IV	3197	16 183		
		Beta turn VIa1	671	1397		
		Beta turn VIa2	215	406		
		Beta turn VIb	1028	2350		
		Beta turn VIII	4874	11 821		
		Gamma turn C	1059	2889		
		Gamma turn I	5833	12 720		
		Beta buldge-B	271	524		
		Beta buldge-C	4926	9214		
		Beta buldge-G	3694	6931		
		Beta buldge-S	717	1304		
		Beta buldge-W	864	1752		
		Hairpin	4931	12 984		
		Psiloop	1197	2460		
3	DNA/RNA-interacting residues	DNA	417	560	7 (1254)	13 (3958)
		RNA	282	415		
4	DNA/RNA-interacting proteins	DNA	417	560	7 (1254)	13 (3958)
		RNA	282	415		
5	Nucleotide-interacting residues	ATP	228	313	4 (605)	22 (9213)
		ADP	300	353		
		GTP	52	83		
		GDP	88	120		
		NAD	133	140		
		FAD	156	172		
		FMN	103	117		
		UDP	51	68		
6	Nucleotide-interacting proteins	ATP	228	313	4 (605)	22 (9213)
		ADP	300	353		
		GTP	52	83		
		GDP	88	120		
		NAD	133	140		
		FAD	156	172		
		FMN	103	117		
		UDP	51	68		
7	Ligand-binding residues	SO4	2604	3312	0 (0)	4 (726)
		PO4	1002	1299		
		NAG	488	727		
		HEM	167	176		
		BME	163	191		
		EDO	1095	1507		
		PLP	64	65		
8	Ligand-binding proteins	SO4	2604	3312	0 (0)	4 (726)
		PO4	1002	1299		
		NAG	488	727		
		HEM	167	176		
		BME	163	191		
		EDO	1095	1507		
		PLP	64	65		
9	Metal-interacting residues	Fe	163	215	0 (0)	9 (1374)
		Mg	1384	1908		
		Ca	1018	1402		
		Mn	386	521		
		Zn	1118	1660		
		Co	149	201		
		Ni	243	355		
10	Metal-interacting proteins	Fe	163	215	0 (0)	9 (1374)
		Mg	1384	1908		
		Ca	1018	1402		
		Mn	386	521		
		Zn	1118	1660		
		Co	149	201		
		Ni	243	355		

^*^Number of non-redundant PDB chains generated using BlastClust at 25% sequence similarity and resolution in between 0–3 Å.

ccPDB 2.0 also allows the user to create its own customized dataset in six simple steps using the `CREATION OF DATASET’ module (See Materials and methods). These customized datasets can be used for developing new method as well as benchmarking other methods. The `WEB SERVICES’ module is another important module which allows the user to analyze its PDB structure as well as annotate it. Analysis of the PDB_ID option of this module comprises only those web services which are functional. We have removed some of the web services in the updated version whose servers were not functional or have been obsolete. We have tried to compile all the possible links of functional standalone software, web services as well as database related to protein structure annotation and function, molecular dynamics and docking in the `IMPORTANT LINKS’ module. We believe that this module will be of great help to all the researchers working in the field of protein structure annotation, function and drug designing. Complete architecture of the ccPDB 2.0 is given in Figure 3.

## Declarations

### Ethics approval and consent to participate

This study does not require any ethical clearance or any consent to participate.

### Availability of data and materials

All data including datasets are freely available to scientific community and can be downloaded from the webserver.

## Authors’ contribution

P.A., S.P., R.K., V.K. and P.K.R. collected and compiled the datasets. P.A. and H.S. performed the experiments. P.A., R.K., V.K.P. and S.P. developed the web interface. P.A. and G.P.S.R. analyzed the data and prepared the manuscript. G.P.S.R. conceived the idea and coordinated the project. All authors read and approved the final paper.

## Notes

**Database URL:** http://webs.iiitd.edu.in/raghava/ccpdb
